# Neighborhood socioeconomic status in relation to mortality rates of gastrointestinal cancers among Asian American, Native Hawaiian, and Pacific Islander populations in California

**DOI:** 10.1007/s10552-026-02261-x

**Published:** 2026-10-06

**Authors:** Austin Lui, Meg McKinley, Abhiraj Sanku, Melody Wang, Maria Lugue, Katherine Ngo, Erin L. Van Blarigan, Iona Cheng

**Affiliations:** 1 College of Osteopathic Medicine, Touro University California, Vallejo, CA USA; 2 Department of Epidemiology and Biostatistics, University of California, San Francisco, San Francisco, CA USA; 3 Department of Urology, University of California, San Francisco, San Francisco, CA USA

**Keywords:** AANHPI populations, Gastrointestinal cancer, Mortality rates, Neighborhood SES

## Abstract

**Objective:**

Gastrointestinal (GI) cancers account for approximately 24% of all cancer deaths in the United States (US). Demographic and socioeconomic status (SES) factors have been associated with mortality in GI cancers. However, the associations between GI cancer mortality and neighborhood SES (nSES) among non-Hispanic Asian American, Native Hawaiian, and Pacific Islander (AANHPI) populations in California have not been investigated. This study examined GI cancer mortality rates by nSES among AANHPI adults in California.

**Methods:**

The California Cancer Registry, part of the population-based Surveillance, Epidemiology and End Results (SEER) program of the National Cancer Institute, was used to identify AANHPI males and females who died of GI cancers from 2008 through 2012. GI cancer deaths were defined by the following sites: colorectum, stomach, esophagus, small intestine, and anus or anal canal. Each death was assigned a nSES index using the 2010 US Census data. Age-adjusted mortality rates of GI cancer and mortality rate ratios (MRR) as well as 95% confidence intervals by nSES quintiles were calculated for a 5-year period from 2008 through 2012. Subgroup analyses were conducted by sex and cancer site.

**Results:**

A total of 5,153 cases were included in the final study population for analysis. Among all AANHPI males and females, those residing in the lowest nSES quintile had a 1.31-fold higher MRR of GI cancers compared to those in the highest nSES quintile (Q1 vs. Q5; 95% confidence interval (CI): 1.19–1.45). This positive association was observed in both males and females, although not statistically significant in females (males: nSES Q1 vs. Q5 MRR: 1.50; 95% CI: 1.31–1.71; females: nSES Q1 vs. Q5 MRR: 1.11; 95% CI: 0.96–1.29). Similar patterns of associations were seen across specific GI cancer sites.

**Conclusion:**

AANHPI adults in California residing in lower nSES neighborhoods experienced higher GI cancer mortality compared with those in higher nSES areas. Future studies are needed to clarify the underlying mechanisms and identify actionable strategies to mitigate nSES-related inequities in GI cancer outcomes in the AANHPI population.

## Introduction

Gastrointestinal (GI) cancers collectively are the most common types of cancer in the United States (US) and are the second leading cause of cancer mortality in the US[1]. Lower individual-level socioeconomic status (SES), measured using income and education, has been associated with higher incidence and mortality of GI cancers and lower treatment rates in GI cancer patients[2–10]. Neighborhood SES (nSES), a composite index capturing multiple area-level domains of SES including population income, occupation, and education level, has also been associated with survival in GI cancer[11–13]. This was highlighted in a study of colorectal cancer (CRC) among the Hispanic population in California (1988–2010) which reported those residing in lower nSES areas had higher CRC mortality rates compared to those residing in higher nSES areas (HR, 1.16 95% CI 1.03–1.30)[12].

According to the National State of Asian American, Native Hawaiian, and Pacific Islanders (AANHPIs) report, the Asian American population grew nearly 40% while the Native Hawaiian and Pacific Islander population grew by nearly 30% between 2000 and 2010, making these populations among the fasting growing in the US[14]. In both AANHPI males and females, CRC is currently the third most common cancer diagnosis, with a majority being diagnosed at an advanced stage[15]. Studies have examined associations between area-level SES and CRC mortality among AANHPI adults[2, 16]. One study utilizing California Cancer Registry data from 1999 through 2001 observed that lower nSES was associated with higher CRC mortality rates in the AANHPI population. Interestingly, a county-level study found that initially between 1990 through 1992, poorer counties had lower mortality rates when compared to the richest counties[2]. However, by 2003–2007, the poorest counties had a higher CRC mortality rate across all sex, racial, and ethnic groups[2]. In contrast, another study utilizing California Cancer Registry data from 1999 through 2001 found no significant association between nSES and CRC mortality among AANHPI adults aged 50 years and older[16].

While studies have examined the association between nSES and CRC mortality rates in the AANHPI population, few have studied the association between nSES and the mortality rates of other subsites of GI tract cancers such as esophageal, stomach, small intestine, and anal cancers. Studies suggest that the AANHPI population face varying disparities in mortality rates depending on the specific GI tract cancer type relative to other racial/ethnic groups. For example, gastric cancer mortality rates are signficantly higher in the AANHPI population when compared to non-Hispanic Whites[17], while esophageal squamous cell carcinoma mortality rates are higher in the AANHPI population relative to non-Hispanic Whites[18].

Neighborhood SES has emerged as an important social determinant of cancer outcomes through its influence on access to screening, timely diagnosis, treatment utilization, and survivorship care. Because GI tract malignancies share common risk factors and healthcare pathways that may be influenced by socioeconomic conditions, evaluating GI tract cancers collectively may provide insight into broader socioeconomic patterns in cancer mortality. However, the relationship between nSES and GI tract cancer mortality among AANHPI populations remains poorly understood. To our knowledge, no studies to date have examined the associations between nSES and overall GI tract cancer mortality and individual GI tract cancer subsites specifically in the AANHPI population in California.

In this report, we examined the associations between nSES and mortality rates from GI cancers among the AANHPI population, by sex and cancer site, using California Cancer Registry (CCR) data collected from 2008 through 2012 linked with vital statistics from the California Department of Public Health. These years were used because our exposure was based on the 2010 Census, which provided the most recent available data for the nSES index at the time this study was conducted.

For this study, we sought to evaluate GI cancers that share similar opportunities for SES effects through screening, surveillance, diagnostic evaluation, and access to specialty care. As such, we examined specifically GI tract cancers deaths, which was defined as deaths from malignancies arising from the GI tract (esophagus, stomach, small intestine, colorectum, anus, and anal canal) and did not include deaths from cancers of other digestive organs such as the pancreas, liver, or gallbladder, which have distinct etiologic and clinical characteristics.

In addition to evaluating individual GI tract cancer subsites, we examined overall GI tract cancer mortality because these malignancies share several common biological and healthcare-related characteristics, including exposure to dietary risk factors[19], reliance on endoscopic diagnostic and surveillance pathways[20], and dependence on access to preventive and specialty care services[20]. Evaluating GI tract cancers deaths collectively allows assessment of whether a broader socioeconomic gradient exists across GI tract malignancies as a whole while also providing greater statistical power than analyses restricted to individual cancer subsites. Site-specific analyses were subsequently performed to determine whether observed associations were consistent across specific GI tract cancer types.

We utilized the CCR, as it is one of the most comprehensive and high-quality population-based cancer registries in the US, with near-complete statewide capture of cancer diagnosis, as required by California state law. California is also home to one of the largest and most diverse AANHPI populations in the US[21]. In addition, California’s broad socioeconomic diversity further enhances the ability to detect contextual disparities in cancer outcomes.

## Methods

### Ascertainment of GI cancer deaths and study population

Using mortality data from the CCR[22], part of the National Cancer Institute’s (NCI) Surveillance, Epidemiology, and End Results (SEER) program, we identified deaths where GI cancer was the underlying cause among non-Hispanic AANHPI males and females in California from 1 January 2008, through 31 December 2012[23]. Using the SEER Cause of Death Recode variable, GI cancer deaths were defined by the following sites: 1) colorectum, 2) stomach, 3) esophagus, 4) small intestine, and 5) anus or anal canal[23]. Cancers of the pancreas, liver, and gallbladder were not included because these malignancies arise from other digestive organs and differ substantially from GI tract cancers with respect to anatomy, mucosal biology, etiologic risk factors, screening practices, and clinical presentation. In particular, GI tract cancers share common dietary exposures and frequently involve endoscopic pathways for screening, diagnosis, and surveillance, whereas pancreatic and hepatobiliary malignancies are characterized by distinct disease-specific risk factors and generally lack established population-level screening strategies[20, 24, 25]. Age of death, cause of death, race and ethnicity, and quintile of nSES were also extracted from the database. From 2008 through 2012, a total of 5,135 AANHPI deaths from GI cancers were included in this study.

This 5-year pericensal period was selected because the appropriate census-tract-level population denominators required for calculation of nSES-stratified mortality rates were available from the 2010 Census. Because annual intercensal population estimates stratified by census tract group, bridged-race, ethnicity, sex, age, and nSES were not available, we used 2010 Census population counts as the population denominator and multiplied these counts by five to estimate the total population at risk during the 2008–2012 study period. This approach is consistent with previously published California Cancer Registry studies examining nSES disparities in cancer outcomes[26, 27]. Bridged-race and ethnicity population estimates refer to Census population counts in which individuals reporting multiple races are reassigned to a single-race category to ensure consistency between mortality numerators and population denominators for rate calculations.

### Measurement of neighborhood socioeconomic status

Patient residential address at diagnosis, as recorded in the CCR, was geocoded to census tracts. Cases were linked to the California Neighborhoods Data System, which provided tract-level measures of nSES derived from U.S. Census and the American Community Survey (ACS) data[28]. Specifically, the nSES composite score was derived using the “Yang Index” censustract-level SES-related variables[29]. This composite measure was developed by Yang et al., which used a similar approach to the composite measure used by Yost et al., and was incorporated into the database prior to analysis. As described by Yang et al. and in the SEER Incidence Data with Census Tract Attributes Database variable definition, the censustract nSES composite score was calculated using factor analysis of SES variables from the ACS, including median household income, median house value, median rent, percent below 150% of poverty line, education index, percent working class, and percent unemployed[29–31]. The 2007–2011 ACS 5-year estimates were used because it provided the most contemporaneous census tract-level socioeconomic data corresponding to the 2010 Census period. Neighborhood SES was categorized into quintiles based on the statewide distribution[31].

### Statistical analysis

Using the NCI’s SEER*Stat software (version 8.4.5) [32], cancer deaths were stratified based on residential address at diagnosis into nSES quintiles (1–5), with 1 as the lowest quintile and 5 as the highest quintile [33]. Deaths were categorized by cancer site: 1) colorectum, 2) stomach, 3) esophageal, 4) small intestine, and 5) anus or anal canal, and collectively as overall GI cancer mortality. Mortality rates, mortality rate ratios (MRR), and 95% confidence intervals (CI) (Tiwari modification) were calculated for the 5-year period, 2008–2012, using those who lived in the highest nSES quintile as the reference group. Neighborhood SES quintile-specific mortality rates were age-adjusted using the direct method and standardized to the 2000 U.S. Standard Population, consistent with NCI and SEER reporting conventions. The numbers of cases for small intestine and anal canal cancers were too few to allow stratification by sex or nSES quintile. Therefore, these subsites were excluded from subgroup analyses.

## Results

### Study population characteristics

**Fig. 1:**
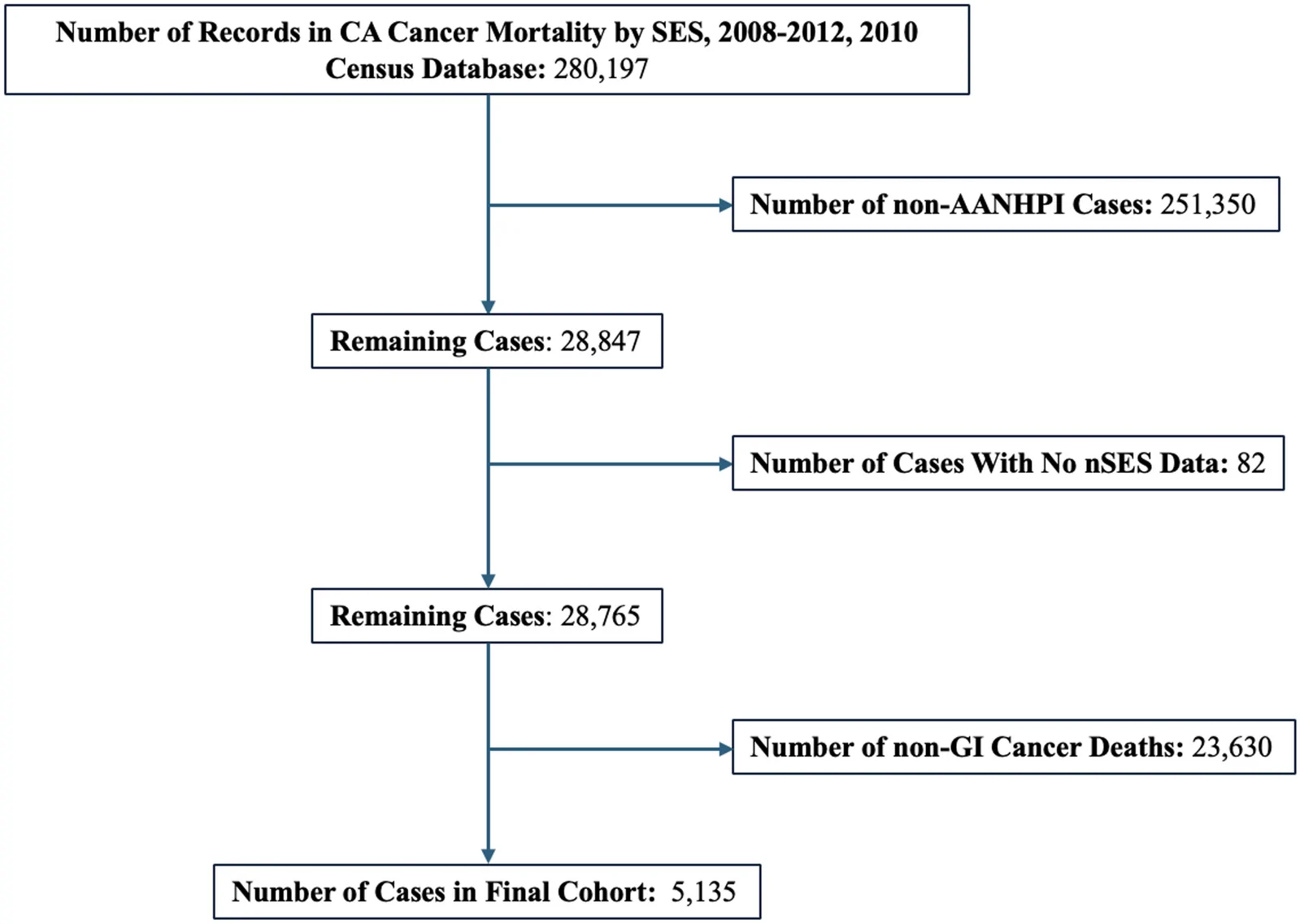
Flow diagram depicting number of cases excluded at each exclusion step to arrive at the final cohort. CA California, SES socioeconomic status, AANHPI Asian American Native Hawaiian, Pacific Islander, nSES neighborhood socioeconomic status, GI gastrointestinal

In the California Cancer Mortality by SES 2008–2012, 2010 Census database, there were a total of 280,197 cases. After excluding non-AANHPI cases, cases with no nSES data, and non-GI cancer deaths, we arrived at a final cohort of 5,153 AANHPI individuals who died from GI cancers in California: 59% with CRC, 31% with stomach cancer, 8% with esophageal cancer, 1% with small intestine cancer, and 0.5% with anus, anal canal, or anorectum cancer (Table 1, Fig. 1). At age of death, 24% were between 70 and 79 years, and 32% were 80 years or older. Additionally, 53% of deaths were male and 47% were female.

**Table 1:** Study characteristics of AANHPI individuals in California who died due to a GI cancer, 2008–2012

	All GI cancers deaths^1^		Colorectal cancer deaths		Stomach cancer deaths		Esophageal cancer deaths		Small intestine cancer deaths		Anal, anal canal, and anorectum cancer deaths	
	n	%	n	%	n	%	n	%	n	%	n	%
Total	5,153	100	3,019	58.6	1,604	31.1	435	8.4	71	1.4	24	0.5
Sex												
Male	2,748	53.3	1,513	50.1	874	54.5	310	71.3	41	57.7	10	0.4
Female	2,405	46.7	1,506	49.9	730	45.5	125	28.7	30	42.3	14	0.6
Age at diagnosis (years)												
<40	123	2.4	70	2.3	44	2.7	^	^	^	^	^	^
40–49	348	6.8	223	7.4	96	6.0	22	5.2	^	^	^	^
50–59	771	15.0	489	16.2	203	12.7	68	15.9	^	^	^	^
60–69	1002	19.4	558	18.5	321	20.0	107	25.1	^	^	^	^
70–79	1244	24.1	694	23.0	403	25.1	117	27.4	25	5.9	^	^
80+	1665	32.3	985	32.6	537	33.5	113	26.5	22	5.2	^	^
nSES												
Quintile 1 (Low)	684	13.3	398	13.2	213	13.3	67	15.4	^	^	^	^
Quintile 2	908	17.7	551	18.3	271	16.9	75	17.3	^	^	^	^
Quintile 3	1,163	22.6	683	22.7	362	22.6	88	20.3	23	5.3	^	^
Quintile 4	1,213	23.6	716	23.8	375	23.4	101	23.3	20	4.6	^	^
Quintile 5 (High)	1,167	22.7	658	21.9	379	23.6	103	23.7	17	3.9	^	^

*AANHPI *Asian American, Native Hawaiian, Pacific Islander, *GI* gastrointestinal, *nSES* neighborhood socioeconomic status

^Value suppressed due to being < 11.

^1^GI Cancers (All) include Esophagus, Stomach, Small Intestine, Colorectal, Anus, and Anal Canal and Anorectum. There were 24 deaths due to anal cancer. These individuals are included in “All GI cancers.”

### Association between nSES and GI cancer mortality rates among AANHPI individuals

Overall, AANHPI individuals residing in areas of the lowest nSES quintile had higher GI cancer mortality rates compared to those residing in the highest nSES quintile (Q1 vs Q5 (ref.) MRR: 1.31; 95% CI: 1.19–1.45) (Table 2). These results appeared to be driven by the association observed among males. Males residing in low nSES areas had 50% higher GI cancer mortality rates compared to males residing in high nSES areas (Q1 vs. Q5 MRR: 1.50; 95% CI: 1.31–1.71). Among females, there was a positive association between nSES and GI mortality, but this was not statistically significant (Q1 vs. Q5 MMR: 1.11; 95% CI: 0.96–1.29).

**Table 2:** Mortality rates and MRRs in the California AANHPI population with GI cancer stratified by nSES and sex, 2008–2012

	Overall			Male			Female		
nSES	Deaths (n = 5,135)	Mortality Rate^1^ (95% CI)	MRR (95% CI)	Deaths (n = 2737)	Mortality Rate^1^ (95% CI)	MRR (95% CI)	Deaths (n = 2398)	Mortality Rate^1^ (95% CI)	MRR (95% CI)
Quintile 5 (High)	1,167	19.41 (18.28–20.60)	1.00 (reference)	602	23.11 (21.2–25.1)	1.00 (reference)	565	16.54 (15.2–18.0)	1.00 (reference)
Quintile 4	1,213	21.53 (20.31–22.8)	1.11 (1.02–1.21)	651	27.63 (25.5–29.9)	1.20 (1.06–1.34)	562	17.24 (15.8–18.7)	1.04 (0.92–1.18)
Quintile 3	1,163	21.87 (20.62–23.18)	1.13 (1.04–1.23)	623	27.8 (25.6–30.1)	1.20 (1.07–1.35)	540	17.54 (16.1–19.1)	1.06 (0.94–1.20)
Quintile 2	908	22.62 (21.16–24.16)	1.17 (1.07–1.27)	463	27.07 (24.6–29.7)	1.17 (1.03–1.33)	445	19.4 (17.6–21.3)	1.17 (1.03–1.33)
Quintile 1 (Low)	684	25.47 (23.58–26.48)	1.31 (1.19–1.45)	398	34.59 (31.3–38.1)	1.50 (1.31–1.71)	286	18.4 (16.3–20.7)	1.11 (0.96–1.29)

Bold values indicate statistical significance (p < 0.05) comparing to the highest quintile nSES group.

*CI* confidence interval, *MRR* mortality rate ratio, *AANHPI* Asian American, Native Hawaiian, Pacific Islander *GI* gastrointestinal, *nSES *neighborhood socioeconomic status

GI Cancers include Esophagus, Stomach, Small Intestine, Colorectal, Anus, and Anal Canal and Anorectum.

^1^Rates are per 100,000 and age-adjusted to the 2000 U.S. Standard Population (19 age groups - Census P25-1130) standard; Confidence intervals (Tiwari mod) are 95% for rates and rate ratios.

### Association between nSES and mortality rates by GI cancer site among AANHPI individuals

Table 3 presents the associations between nSES, sex, and mortality rates for GI cancers in AANHPI individuals by cancer site: CRC, stomach, and esophagus. For each of the three cancer sites examined, residing in a low nSES area was associated with higher mortality than residing in a high nSES area. The MRR (95% CI) for Q1 vs. Q5 (ref) were as follows: 1.37 (1.21–1.56) for CRC, 1.23 (1.03–1.47) for stomach, and 1.47 (1.06–2.04) for esophageal cancer. Moreover, similar patterns of associations were observed by sex, whereby the associations seemed to be stronger among males than among females.

**Table 3:** Mortality rates and MRRs in the California AANHPI population with GI cancer by cancer subtypes, stratified by nSES and sex, 2008–2012

Site	Overall			Male			Female		
	Deaths	Mortality Rate^1^ (95% CI)	MRR (95% CI)	Deaths	Mortality Rate^1^ (95% CI)	MRR (95% CI)	Deaths	Mortality Rate^1^ (95% CI)	MRR (95% CI)
Colorectal									
Quintile 5	658	10.82 (9.98–11.71)	1.00 (reference)	314	11.74 (10.41–13.18)	1.00 (reference)	344	10.08 (9.02–11.24)	1.00 (reference)
Quintile 4	716	12.7 (11.77–13.38)	1.17 (1.05–1.31)	369	15.66 (14.05–17.39)	1.33 (1.14–1.57)	347	10.61 (9.51–11.81)	1.05 (0.9–1.23)
Quintile 3	683	12.73 (11.78–13.74)	1.18 (1.05–1.31)	348	15.31 (13.71–17.05)	1.3 (1.11–1.53)	335	10.8 (9.67–12.04)	1.07 (0.92–1.25)
Quintile 2	551	13.73 (12.6–14.95)	1.27 (1.13–1.42)	258	15.07 (13.26–17.06)	1.28 (1.08–1.53)	293	12.75 (11.32–14.33)	1.26 (1.08–1.49)
Quintile 1	398	14.87 (13.43–16.43)	1.37 (1.21–1.56)	216	18.85 (16.4–21.57)	1.61 (1.34–1.93)	182	11.68 (10.01–13.56)	1.16 (0.96–1.40)
Stomach									
Quintile 5	379	6.45 (5.8–7.15)	1.00 (reference)	205	8.36 (7.22–9.63)	1.00 (reference)	174	5.01 (4.28–5.84)	1.00 (reference)
Quintile 4	375	6.72 (6.04–7.45)	1.04 (0.9–1.21)	195	8.49 (7.3–9.8)	1.01 (0.82–1.25)	180	5.52 (4.73–6.4)	1.1 (0.89–1.37)
Quintile 3	362	6.9 (6.2–7.65)	1.07 (0.92–1.24)	195	8.93 (7.69–10.29)	1.07 (0.87–1.31)	167	5.46 (4.66–6.36)	1.09 (0.87–1.36)
Quintile 2	271	6.79 (6–7.66)	1.05 (0.9–1.24)	148	8.79 (7.41–10.35)	1.05 (0.84–1.31)	123	5.38 (4.47–6.44)	1.07 (0.84–1.37)
Quintile 1	213	7.94 (6.9–9.1)	1.23 (1.03–1.47)	128	11.18 (9.32–13.32)	1.34 (1.06–1.68)	85	5.56 (4.42–6.92)	1.11 (0.84–1.46)
Esophagus									
Quintile 5	103	1.67 (1.35–2.03)	1.00 (reference)	73	2.54 (1.97–3.22)	1.00 (reference)	30	0.94 (0.63–1.35)	1.00 (reference)
Quintile 4	101	1.73 (1.4–2.11)	1.04 (0.77–1.39)	73	2.84 (2.21–3.61)	1.12 (0.79–1.59)	28	0.89 (0.59–1.3)	0.95 (0.54–1.66)
Quintile 3	88	1.67 (1.33–2.06)	1 (0.74–1.35)	60	2.66 (2.01–3.44)	1.05 (0.72–1.51)	28	0.94 (0.62–1.37)	1 (0.57–1.75)
Quintile 2	75	1.82 (1.42–2.29)	1.09 (0.79–1.49)	52	2.91 (2.16–3.84)	1.15 (0.78–1.68)	23	1.00 (0.63–1.52)	1.06 (0.59–1.93)
Quintile 1	67	2.46 (1.9–3.13)	1.47 (1.06–2.04)	52	4.38 (3.26–5.77)	1.72 (1.18–2.52)	^	~	~

Bold values indicate statistical significance (p < 0.05) comparing to the highest quintile nSES group.

*CI* confidence interval, *MRR *mortality rate ratio, *AANHPI *Asian American Native Hawaiian, Pacific Islander, *GI* gastrointestinal, *nSES* neighborhood socioeconomic status

~Statistic could not be calculated.

^Value suppressed due to being < 16.

^1^Rates are per 100,000 and age-adjusted to the 2000 US Standard Population (19 age groups - Census P25-1130) standard; Confidence intervals (Tiwari mod) are 95% for rates and rate ratios.

## Discussion

In this study, among AANHPI adults residing in California with deaths from a GI cancer from 2008 through 2012, we observed that living in lower nSES areas was associated with higher mortality rates of GI cancers. This association was more pronounced among males than females. Recognizing the heterogeneity of each individual site-specific GI malignancies, we also performed site-specific analyses to evaluate whether associations were consistent across specific cancer types. While a clear nSES gradient was observed for overall GI tract cancer mortality and CRC mortality, associations were less consistent for gastric and esophageal cancers. In particular, while the largest mortality rate ratio comparing the lowest and highest nSES quintiles was observed for esophageal cancer, CRC demonstrated the most consistent nSES gradient, with significantly elevated mortality rates observed across all lower nSES quintiles relative to the highest nSES quintile. Like esophageal cancer, the only significantly higher gastric cancer mortality rate ratio was comparing the lowest to the highest nSES quintile, although this was not as strong of an association compared to the one found in esophageal cancer cases. These differences may reflect underlying heterogeneity between cancer sites and/or reduced statistical power due to the smaller number of gastric and esophageal cancer cases.

The relatively large number of CRC deaths observed in our study may partially reflect the burden of CRC within the AANHPI population. Previous studies have demonstrated that CRC is among the most common GI malignancies in several AANHPI subgroups, although important heterogeneity exists across populations[34]. Consequently, higher CRC mortality rates may in part reflect higher underlying disease incidence in addition to potential differences in socioeconomic factors, screening utilization, and access to care.

A previous study by Kish et al. utilizing SEER registries across the US from 2002 through 2008 found that AANHPI patients with CRC residing in the lowest nSES quintile had a significantly higher CRC-specific mortality rate compared to those in the highest nSES quintile (HR = 1.36, p < 0.05)[35]. Our findings are consistent with these results and further support the presence of nSES disparities in CRC mortality among AANHPI populations. Together, these studies suggest that lower nSES is associated with worse CRC outcomes in AANHPI individuals across diverse geographic settings.

Because lower CRC screening rates are well established in the literature to be associated with higher CRC-specific mortality[36], it is plausible that the observed association between lower nSES and increased mortality in our study may be partly explained by reduced screening uptake among lower nSES groups. Consistent with this hypothesis, the 2009 California Health Interview Survey found that AANHPI individuals at 0–99% federal poverty level (FPL) had a CRC screening compliance of 10.9% compared to 57.7% compliance in those at 300% FPL and above. Lower CRC screening adherence among individuals in low nSES neighborhoods is likely driven by multiple intersecting barriers, including reduced access to healthcare services, underinsurance or lack of insurance, greater economic and logistical constraints, and lower levels of health literacy.

Esophageal and stomach cancer mortality rates in the AANHPI population have not been previously described according to nSES. Our findings showed that esophageal and stomach cancers also have statistically significant higher mortality rates among AANHPI individuals in low nSES groups. This extends the nSES gradient observed in CRC to other GI cancers and highlights the broad influence of neighborhood context on AANHPI GI cancer outcomes. These disparities may reflect delayed diagnosis due to limited access to specialty care and endoscopy[37], differences in access to specialized cancer care[37], language and navigation barriers[38], and underlying differences in risk factor prevalence or management, such as H. pylori infection and early-life exposures[39], across nSES levels. Further research is needed to clarify these relationships and to investigate the mechanisms driving nSES-related disparities in these less common GI cancers within AANHPI communities.

In our study, we observed a difference by sex when examining the associations between nSES and GI cancer mortality rates in AANHPI California residents. Overall, males from lower nSES areas had higher overall GI cancer mortality and site-specific GI cancer mortality rates when compared to males residing in the highest nSES areas. Although similar patterns were present in females, the associations were weaker. While these differences may partly reflect the smaller number of female cases and reduced statistical power, they may also represent true underlying social or behavioral differences. Consistent with our findings, Singh et al. reported stronger associations between area-level socioeconomic deprivation and county-level, age-adjusted cancer mortality rates in males than in females[40]. Such discrepancies may be driven by sex-specific variations in screening uptake[41, 42], stage at diagnosis[43], comorbidity burden[43], health-seeking behaviors[41, 42], and access to timely or high-quality treatment[41, 42], all of which could influence the strength of the nSES and mortality relationship.

It is also possible that females may have a different social support system compared to males. A study by Kcomt and Gorey found that Chinese American females were more likely than males to live in enclaves, defined as neighborhoods where 10% or more residents were Chinese Americans[8]. Moreover, this study found that Chinese American females who lived in enclaves had a significantly large colon cancer survival advantage, while this survival advantage was not found in Chinese American males[8]. Thus, social support and resources may differ between males and females, which may be one factor contributing to differences in the observed associations between nSES on GI cancer mortality rates.

Future research on AANHPI GI cancers by nSES should aim to identify specific social and built-environment factors that underlie the observed mortality gradients. Beyond screening disparities, neighborhood conditions such as access to high-quality primary and specialty care, availability of gastroenterology and oncology services, transportation infrastructure, linguistic isolation, housing instability, and exposure to environmental stressors may shape timely diagnosis and treatment[44–47]. Additionally, although prior studies have explored sex differences in how SES influences cancer outcomes, the mechanisms through which sex interacts with neighborhood context to affect GI cancer mortality remain poorly understood and warrant further investigation. A deeper examination of these multilevel determinants may clarify the pathways through which neighborhood disadvantage contributes to excess mortality in AANHPI populations.

## Limitations and considerations

We used the California Cancer Registry data to identify GI cancer deaths among non-Hispanic AANHPI females and males in California from 1 January 2008, through 31 December 2012. With this older dataset, it is important to conduct follow-up studies using contemporary data to corroborate our findings. However, the findings from this study are still informative given the limited studies to date on the associations between nSES and GI cancer mortality among the AANHPI population. Our mortality rate calculations relied on 2010 Census population counts multiplied across the 5-year study period because annual intercensal population estimates at the census tract level were unavailable. Although the study period was centered on the 2010 Census, population growth and demographic shifts among AANHPI populations may have resulted in some imprecision in denominator estimates and, consequently, mortality rate calculations. In particular, if population estimates have increased over time, the size of the denominator estimates may be underestimated and therefore overestimate the morality rates in the later years of our analysis. It was also not possible to disaggregate into more detailed categories of AANHPI ethnicities. Therefore, we were not able to perform subgroup analysis to identify nSES and mortality associations among various AANHPI subpopulations.

Additionally, information on tumor stage, histologic grade, and cancer treatment was not available for this analysis. These factors are important for cancer mortality and may differ across socioeconomic contexts. In particular, individuals with low nSES may face barriers to screening, timely diagnosis, specialty referral, and treatment access, potentially resulting in more advanced disease at presentation and differences in treatment utilization. Future studies incorporating detailed clinical and treatment data are needed for prospective epidemiologic studies to investigate the contribution of clinical and socioeconomic factors on outcomes for GI cancers among AANHPI populations.

We were also unable to examine differences by urban versus rural residence because urban/rural classification was not available in the analytic dataset. This may be relevant because geographic context can influence access to screening, diagnostic evaluation, specialty care, and treatment resources. Future studies incorporating measures of urbanicity may help clarify whether nSES disparities in GI cancer mortality differ across geographic settings. Furthermore, although nSES was taken into account for our analysis, another limitation was not being able to adjust for individual-level SES. Finally, there were limited number of cases in certain GI cancer subtypes, such as small intestine and anal cancers, which precluded any further stratification of these GI cancer subtypes by nSES quintiles and subsequent analysis.

## Conclusion

In this study, we found that lower nSES was associated with higher GI cancer mortality among AANHPI individuals in California, with more pronounced associations observed in males than females. Although the largest mortality rate ratio comparing the lowest and highest nSES quintiles was observed for esophageal cancer, colorectal cancer demonstrated the most consistent socioeconomic gradient across the entire nSES strata. These findings highlight the importance of neighborhood context in shaping cancer outcomes within the AANHPI population. Future research should aim to identify the specific social and built-environment mechanisms that drive these disparities. A deeper understanding of these pathways will be essential for developing targeted interventions to reduce preventable mortality and promote equitable cancer outcomes among AANHPI populations.

## Data Availability

The dataset analyzed during the current study are derived from the California Cancer Registry, part of the population-based Surveillance, Epidemiology and End Results (SEER) program of the National Cancer Institute. Inquiries regarding access to CCR data can be made here: [https://www.ccrcal.org/retrieve-data/data-for-researchers/] (https:/www.ccrcal.org/retrieve-data/data-for-researchers).
